# 7.C. Workshop: Towards a true European Health Union: milestones, drivers and barriers

**DOI:** 10.1093/eurpub/ckac129.408

**Published:** 2022-10-25

**Authors:** Organised by: European Observatory on Health Systems and Policies, Chair persons: Matthias Wismar (European Observatory)

## Abstract

European health system responses to the COVID-19 pandemic have also demonstrated that European integration in health and health systems add value beyond what individual Member States can do: ranging from the joint purchase of medicines to providing large budgets for building back better. With a true European Health Union we will strengthen our health systems beyond crisis preparedness and response, which is necessary as we are facing the evolving perma-crisis, We will need to strengthen our health systems in general always with a view to Universal Health Coverage and improving health system performance. The European Health Union can work to help strengthen health systems, which may include support for Member States research, learning from best practices, investment but also common solutions including new or extended EU mandates, new arms-length bodies, as well as more coordination or harmonization. It has also taught us that we cannot wait to strengthen our health systems as we are in Europe in a perma-crisis.

The panel will address the following questions:

• Where are we with our progress towards a true European Health Union?

• What policy change is needed to create a true European Health Union?

• What would be the role of the true European Health Union strengthening and supporting Member States?

We will zoom in on the 1) existing public health mandate of the EU, 2) the impact of other EU-policies on health and health systems, 3) the new economic governance and, 4) the role of the EU in global public health

**Key messages:**

• A true European Health Union will strengthen universal health coverage and health system performance.

• A true European Health Union goes beyond crisis-preparedness and respons.

**Speakers/Panellists:**

**Scott Greer**

University of Michigan, European Observatory on Health Systems and Policies, Ann Arbor, USA

**Anniek de Ruijter**

University of Amsterdam, Amsterdam, Netherlands

**Eleanor Brooks**

University of Edinburgh, Edinburgh, UK

**Thibaud Deruelle**

University of Lausanne, Lausanne, Switzerland

